# Therapeutic targeting of VEGF and/or TGF-β to enhance anti-PD-(L)1 therapy: The evidence from clinical trials

**DOI:** 10.3389/fonc.2022.905520

**Published:** 2022-07-26

**Authors:** Linwei Li, Qinglian Wen, Ruilin Ding

**Affiliations:** ^1^ Department of Oncology, Affiliated Hospital of Southwest Medical University, Luzhou, China; ^2^ Institute of Drug Clinical Trial/GCP Center, Affiliated Hospital of Southwest Medical University, Luzhou, China

**Keywords:** tumor microenvironment, VEGF, TGF-β, immunotherapy, PD-1, review

## Abstract

Normalizing the tumor microenvironment (TME) is a potential strategy to improve the effectiveness of immunotherapy. Vascular endothelial growth factor (VEGF) and transforming growth factor (TGF)-β pathways play an important role in the development and function of the TME, contributing to the immunosuppressive status of TME. To inhibit VEGF and/or TGF-β pathways can restore TME from immunosuppressive to immune-supportive status and enhance sensitivity to immunotherapy such as programmed death protein-1 (PD-1)/programmed cell death-ligand 1 (PD-L1) inhibitors. In this review, we described the existing preclinical and clinical evidence supporting the use of anti-VEGF and/or anti-TGF-β therapies to enhance cancer immunotherapy. Encouragingly, adopting anti-VEGF and/or anti-TGF-β therapies as a combination treatment with anti-PD-(L)1 therapy have been demonstrated as effective and tolerable in several solid tumors in clinical trials. Although several questions need to be solved, the clinical value of this combination strategy is worthy to be studied further.

## Introduction

The tumor microenvironment (TME) is a complex environment characterized by several unique properties including hypoxia, a low PH, an abnormal tumor immune microenvironment (TIME), a high interstitial fluid pressure and vascular abnormalities (1). The treatments targeting TME have been proposed as the promising strategies for cancer treatments, especially immunotherapy and antiangiogenic therapy (1, 2).

Recent years, immunotherapy has shown dramatic effects in the management of various human cancers. The programmed death protein-1 (PD-1)/programmed cell death-ligand 1 (PD-L1) pathway mediates tumor immune evasion. The agents targeting PD-(L)1 have the potential to reverse the inhibition of cytotoxic T cells in the TME, exhibiting promising antitumor activity in various tumor types (2–4). However, 50-80% of patients with tumors do not benefit from PD-(L)1 inhibitors as monotherapies (5). TME has been reported to reduce the effectiveness of immunotherapy (6). Thus, normalizing the TME is regarded as a promising strategy to enhance immunotherapy.

Previous studies have revealed that overexpression of vascular growth factors inhibited immune effector cells and activated immunosuppressive cells directly (1, 5). And the hypoxia TME caused by abnormal tumor vascular structure can prevent immune cell infiltration (1). Thus, tumor vascular structure and vascular growth factors such as vascular endothelial growth factor (VEGF) and its receptor (VEGFR), contributed to the immunosuppressive features of TME (7). Currently, VEGF/VEGFR2 pathway is the predominant target for the development of anti-angiogenesis agents. The efficacy of the combination of anti-PD-(L)1 and anti-VEGF has been evaluated in clinical trials (8, 9).

As a well-studied pleiotropic cytokine, transforming growth factor (TGF)-β plays a vital role in regulating cellular proliferation. Three TGF-β isoforms have been identified in mammals: TGF-β1 (the most common type), TGF-β2, and TGF-β3 (10). In this review, we mainly discussed the function role of TGF-β1. TGF-β in TME has been identified to promote tumor cell invasiveness, migration, and metastasis through mechanisms including tumor angiogenesis and epithelial-mesenchymal transition (EMT) (11). High expression of TGF-β in the TME is reported to be associated with poor clinical outcome (12). TGF-β also has immune-related activities, which works to inhibit cytotoxic T cells, induct peripheral Treg from naive CD4^+^ cells and increase the survival of myeloid progenitors that differentiate to potent myeloid-derived suppressor cell (MDSCs) (11–13). The activity of TGF-β is reported to promote resistance to anti-PD-L1 therapies (14, 15). Furthermore, TGF-β in TME has been shown to work cooperatively with hypoxia-inducible factor (HIF)-1, which is increased under hypoxic conditions, in inducing the expression of VEGF, driving tumor angiogenesis (16). Thus, inhibition of the TGF-β pathway has been report to overcome immunosuppression, suppress angiogenesis and EMT, and inhibit tumor growth (12).

Previous data supported the hypothesis that anti-PD-(L)1 combined with anti-VEGF and/or anti-TGF-β may offer stronger antitumor activity by synergistically reducing angiogenesis and further enhancing the immune response against malignancy. Therefore, in the present review, the existing evidence supporting the applicability of this treatment strategy was summarized.

## Rational for targeting VEGF/VEGFR and/or TGF-β for enhancing anti-PD-(L)1 response

### The role of VEGF pathway in TIME

The role of VEGF pathways in TIME has been extensively investigated in tumor models (5). The mechanisms how excessive levels of VEGF induce immunosuppression of TME include at least the following four distinct aspects.

First, VEGF could inhibit dendritic cell (DC) maturation and antigen presentation. DCs are considered as professional and most powerful antigen presenting cells (APCs), which could active T cells and enhance the immune response (17). As early as 1998, Oyama et al. found that VEGF inhibited DC maturation through integrating with its receptor on DC surface (18). Alfaro et al. pointed out that VEGF could inhibit the differentiation of monocytes into DCs (19). Furthermore, Curiel and colleagues found that VEGF can upregulate the expression of PD-L1 on DCs, inhibiting DC antigen presentation function and suppressing the T cells activation (20).

Second, excessive levels of VEGF in TME directly inhibits cytotoxic T lymphocytes (CTL) effector function. The study conducted by Ohm et al. showed an inhibiting effect of VEGF on hematopoietic progenitor cells differentiating to CD8^+^ and CD4^+^ T cells, which contributes to tumor-associated immune deficiencies (21). Voron et al. demonstrated that VEGF produced in TME can enhance the expression of PD-1 and other checkpoints involving in CD8^+^ T cell exhaustion (22). And the treatment targeting VEGF was found to remarkably enhance CD8^+^ T cell effector function (23, 24).

Third, the excessive levels of VEGF can promote the recruitment of immunosuppressive cells. For example, Ning et al. enrolled 36 patients with renal cell carcinoma (RCC) and observed that VEGF expression was positively associated with the frequency of tumor-infiltrating regulatory T-cells (Tregs) (25). Besides, Terme et al. found that VEGF directly triggers Treg proliferation and anti-VEGF therapy, exerting a promising immunologic effect (26). Courau et al. pointed out that silencing of VEGF resulted in dramatically decreased Tregs and MDSCs in tumor infiltrates, markedly improving the efficacy of anti-PD-1 treatment (13).

Fourth, VEGF has an ability to promote adhesion molecules and chemokines expressions, resulting in abnormal tumor vasculature structures, which can lead to a hypoxia and low PH condition in TME, subsequently, forming a selective immune-cell barrier to reduce the infiltration of different immune cells (1, 27).

Collectively, overexpression of VEGF in TME has a role on suppressing immune effector cells and activating immunosuppressive cells. And VEGF-induced tumor vessels has abnormal vascular structure which can prevent immune cell infiltration. Through these mechanisms, VEGF contributes to an immunosuppressive TME.

### The role of TGF-β in TIME

TGF-β is pleiotropic cytokine, produced by activated macrophages, platelets, keratinocytes and fibroblasts (28). TGF-β can regulate fibrosis, EMT, and angiogenesis in tumor growth, it can also exert effect in both innate immune and adaptive immune systems, particularly in immunosuppression (12). The innate immune system is composed by monocytes, macrophages, DCs, granulocytes, and NK cells (29). In the TGF-β-rich TME, the antigen-presenting DCs was reported to shift into a tolerogenic phenotype, with decreased ability to activate T cells (30, 31). The development and differentiation of NK cells is also strongly influenced by TGF-β. Marcoe et al. pointed out that the TGF-β pathway was responsible for NK cell immaturity (32). Viel et al. demonstrated that TGF-β could inhibit the activation of NK cells by inhibiting the mTOR related pathways (33). In myeloid cells, TGF-β was reported to stimulate the survival of monocytes (34). And the inhibition of TGF-β signaling could decrease MDSC differentiation and increase differentiation to proinflammatory macrophages (34). Furthermore, the TGF-β in TME may suppress the inflammatory functions of macrophages, contributing to the immune evasion of cancer cells (35).

In adaptive immune system, TGF-β has been reported to inhibit T cell proliferation, activation, and effector functions. Early studies revealed that TGF-β could inhibit the expression of T-box transcription factor (T-bet) to block Th1 and CD8+ cytotoxic T cells differentiation from naïve T cell (36). Furthermore, Thomas et al. found that TGF-β could specifically inhibit CTL-mediated tumor cytotoxicity through decreasing the levels of cytolytic gene products such as perforin, Fas ligand, granzyme A, granzyme B, and IFNγ (37). And neutralization of systemic TGF-β in mice could restore these cytotoxic gene expression in CTLs, reinforcing tumor clearance (37, 38). These findings suggested an important role of TGF-β pathway in inhibiting tumor antigen-specific T cell priming (38). In addition, Ahmadzadeh and colleague found that TGF-β attenuated the effector function of antigen-specific CD8+ cells obtained (39). TGFβ-rich TME can promote T cells differentiating to a Treg phenotype (40).

Overall, TGF-β in TME acts as an important suppressor of the innate and adaptive immune responses. Targeting TGF-β has been regarded as a novel strategy to enhance the effect of immunotherapy.

### The role of VEGF and/or TGF-β in enhancing the sensitivity to anti-PD-(L)1 therapy

Overexpression of VEGF in TME can inhibit immune effector cells and activate immunosuppressive cells, driving immunosuppression in the TME. Blockade of VEGF/VEGFR pathway may enhance the efficacy of anti-PD-(L)1 agents. The data from a number of preclinical studies supported this hypothesis (41–43). And the efficacy and safety of this combination therapy has also been confirmed in several tumor types in clinical trials.

In a previous study, Courau et al. found that blocking of TGF-β pathway could reduce the number of Treg cells, increase the activity of effector T cells, thus restoring the sensitivity to anti-PD-L1 therapy (13). Similarly, Mariathasan et al. found that TGF-β could shape the TME to restrain anti-tumor immunity by restricting T-cell infiltration (44). Therapeutic co-administration of anti-TGF-β to anti-PD-L1 could facilitate T-cell penetration into the center of tumor and enhance anti-tumor immunity (44). These mechanisms provide basis for the combination of anti-TGF-β and anti-PD-(L)1 therapies.

The cross-talk between VEGF and TGF-β pathways has also been identified previously (45). VEGF and TGF-β are usually co-expressed in tumor tissues in which angiogenesis occurs (46). The results from previous studies have shown that TGF-β up-regulated the expression of VEGF (47). Due to the cross-talk between VEGF and TGF-β pathways, the dual inhibition of VEGF and TGF-β may have a stronger anti-angiogenesis effect. Furthermore, the dual inhibition of TGF-β and VEGF signaling can synergistically reduce the number of Tregs and restore sensitivity to anti-PD-1 treatment (13). Thus, the triple inhibition of VEGF/VEGFR, TGF-β and PD-(L)1 seems rational.

## Clinical trials about the dual inhibition of VEGF/VEGFR and PD-(L)1

### Clinical trials on RCC

IMmotion151 (NCT02420821) was a phase III trial to compare the efficacy of atezolizumab (an anti-PD-L1 agent) plus bevacizumab versus sunitinib in untreated metastatic RCC (mRCC) (48). This trial found that the atezolizumab plus bevacizumab arm had a significantly longer median progression-free survival (PFS) comparing to the sunitinib arm in the PD-L1 positive patient population (11.2 vs. 7.7 months, hazard ratio [HR]=0.74 95% CI: 0.57-0.96, p=0.0217). BTCRC-GU14-003 (NCT02348008) was a phase Ib/II trial to evaluate the potential use of pembrolizumab (a PD-1 inhibitor) combined with bevacizumab in mRCC (49). In the phase II, 48 mRCC patients who were treatment naïve were enrolled. The objective response rate (ORR) of this study was 60.9% (95%CI: 45.4%-74.9%) and the median PFS was 20.7 months (95%CI: 11.3-27.4 months), meeting its primary end point. Thus, the combination of pembrolizumab and bevacizumab is active in mRCC patients as first and subsequent lines of therapy (49).

Axitinib is an oral, potent, small molecule inhibitor of VEGFR 1-3 (50). NCT02853331 was a phase Ib trial that evaluated axitinib in combination with avelumab (an anti-PD-L1 agent) in advanced RCC patients (51). At data cutoff date, 58% of patients achieved complete response (CR)/partial response (PR), showing promising anti-tumor effect (51). Due to these results, a phase III trial (NCT02684006) was conducted. Untreated advanced RCC patients received the treatments of avelumab plus axitinib or the standard-of-care (SOC) sunitinib in a 1:1 ratio at random (52). In PD-L1 positive population, median PFS was 13.8 months vs 7.2 months favoring the avelumab plus axitinib treatment (HR=0.61, 95%CI: 0.47-0.79, p < 0.001). In the overall population, avelumab plus axitinib also resulted in a longer median PFS (13.8 months vs. 8.4 months, HR=0.69, 95%CI: 0.56-0.84; p<0.001) (52). According to these results, the combination of avelumab and axitinib for first-line treatment of clear cell RCC (ccRCC) has been approved by the US Food and Drug Administration (FDA). 861 ccRCC patients received pembrolizumab plus axitinib or sunitinib in a phase III trial named KEYNOTE-426 (NCT02853331) (53). Median PFS was much longer in patients received pembrolizumab plus axitinib than those received sunitinib (15.4 months vs. 11.1 months, HR=0.71, 95%CI: 0.60-0.84, p<0.001). This study demonstrated that pembrolizumab plus axitinib could be used as first-line treatment for ccRCC (53).

Lenvatinib is a multitargeted tyrosine kinase inhibitor (TKI) that mainly inhibits VEGFR1-3 (54). KEYNOTE-146 was a Ib/II study to evaluate lenvatinib combined with pembrolizumab in advanced solid tumors. In the RCC cohort, the ORR at week 24 was 63% (19/30, 95%CI: 43.9%-80.1%), showing encouraging anti-tumor activity in this type of patients (55). In a phase III trial (NCT02811861), 1069 RCC patients were assigned to receive lenvatinib in combination with pembrolizumab, lenvatinib in combination with everolimus, or sunitinib (56). The median PFS was much longer in lenvatinib plus pembrolizumab arm than in sunitinib arm (23.9 months vs. 9.2 months, HR=0.39, 95%CI: 0.32-0.49, P<0.001) (56).

Cabozantinib, is an oral VEGFR1-3 inhibitor. The CheckMate 9ER trial (NCT03141177) compared the efficacy and safety of nivolumab plus cabozantinib and sunitinib in previously untreated advanced RCC (57). Nivolumab plus cabozantinib showed significant benefits over sunitinib in advanced RCC, with median PFS of 16.6 months versus 8.3 months (HR=0.51, 95%CI:0.41-0.64, p<0.001) (57). Furthermore, the combination of cabozantinib with atezolizumab also demonstrated efficacy in patients with advanced RCC in a phase Ib trial (COSMIC-021, NCT03170960) (58).

Famitinib is a multitargeted TKI that mainly against VEGFR, C-Kit, and PDGFR. Famitinib plus camrelizumab (an anti-PD-1 antibody) showed enduring antitumor activity in patients with advanced RCC in a phase II trial (59). The ORR was 84.6% in treatment-naïve patients and 48.0% in pretreated patients, respectively (59). Pazopanib is also a TKI that mainly against VEGFR1-3 (1). Checkmate016 (NCT01472081), a phase I trial, evaluated the efficacy and safety of nivolumab (an anti-PD-1 agent) plus pazopanib or sunitinib in mRCC (60). The results showed that the ORR was 55% in nivolumab + sunitinib arm and 45% in nivolumab + pazopanib arm. For safety, this study showed that the combination of sunitinib or pazopanib and nivolumab caused a high incidence of toxicities which limited the future use of either combination regimen (60). Similarly, a phase I/II study showed that pazopanib plus pembrolizumab had significant hepatotoxicity, despite preliminary signs of efficacy were shown (61).

### Clinical trials on non-small-cell lung cancer (NSCLC)

IMpower150 (NCT02366143) was a phase III clinical trial that randomized metastatic non-squamous NSCLC patients to receive atezolizumab plus carboplatin + paclitaxel (ACP), bevacizumab + carboplatin + paclitaxel (BCP), or atezolizumab + BCP (62). The results showed that adding atezolizumab to BCP significantly improved PFS and OS among patients with non-squamous NSCLC (62).

Dual inhibition of VEGFR2 with ramucirumab and PD-1 with pembrolizumab has shown potential anti-tumor effect in NSCLC by JVDF trial (NCT02443324) (63). The study showed that the ORR was 56.3% and 22.2% for patients with high and lower levels of PD-L1 expression, respectively (63). JVDJ (NCT02572687) was a phase Ia/b trial evaluating the safety and efficacy of ramucirumab and durvalumab (an anti-PD-1 antibody) in multi-tumor types (64). In the NSCLC cohort, the ORR, median PFS and OS were, respectively, 11%, 2.7 and 11 months. More prolonged survival could be obtained from patients with high PD-L1 expression (64).

Anlotinib is a novel TKI that mainly targets VEGFR 1-3, epidermal growth factor receptor (EGFR) and FGFR 1-4. A phase Ib trial (NCT03628521) assessed sintilimab (an anti-PD-1 antibody) and anlotinib in the frontline setting for patients with NSCLC, and found that this combination treatment represented a novel chemotherapy-free regimen for unresectable NSCLC without EGFR/ALK/ROS1 mutations, with an ORR of 72.7% and median PFS of 15 months (65). Another phase Ib trial evaluated the combination of anlotinib and PD-1 inhibitors camrelizumab for advanced NSCLC. Anlotinib plus camrelizumab showed promising efficacy for NSCLC, with a median PFS of 8.2 months and a median OS of 12.7 months (66).

### Clinical trials on hepatocellular carcinoma (HCC)

IMbrave150 (NCT03434379) was a global, open-label, phase III trial, which assigned unresectable HCC patients to receive either atezolizumab in combination with bevacizumab or sorafenib in a 2:1 ratio (67). In this study, atezolizumab plus bevacizumab arm showed longer OS and PFS than sorafenib arm, as the HR for death was 0.58 (95%CI: 0.42-0.79, p<0.001) and HR for PFS was 0.59 (95%CI: 0.47-0.76, p<0.001) (67). ORIENT-32 (NCT0379440) was a phase II-III study to evaluate sintilimab plus bevacizumab biosimilar IBI305 versus sorafenib as a first-line treatment for unresectable HCC (68). In the phase III part, patients in the sintilimab-IBI305 arm had a much longer median PFS (4.6 months vs 2.8 months, HR=0.56, 95%CI: 0.46-0.70, p<0.0001) and OS (median not reached vs 10.4 months, HR=0.57, 95%CI: 0.43-0.75, p<0.0001) than those in the sorafenib arm (68). Furthermore, this combination regimen showed an acceptable safety profile (68).

A phase Ib trial (NCT03006926) had tried to use lenvatinib plus pembrolizumab in the treatment of unresectable HCC (69). This combination showed promising antitumor activity in HCC, with an ORR of 36.0% and median OS of 22 months (69). And the toxicities of this combination treatment were manageable, with no unexpected safety signals (69). Apatinib is a small molecule VEGFR-2-TKI and camrelizumab is an anti-PD-1 antibody. A phase II study (NCT03463876) showed that camrelizumab plus apatinib had promising efficacy in advanced HCC, with ORR of 34.3% (24/70) in the first-line setting and 22.5% (27/120) in the second-line setting, respectively (70).

### Clinical trials on other solid tumors

In addition to RCC, NSCLC and HCC, clinical trials about dual inhibition of VEGF/VEGFR and PD-(L)1 in other types of solid tumors are also abundant.

For instance, lenvatinib plus pembrolizumab showed potential antitumor activity in patients with advanced gastric cancer or advanced endometrial carcinoma (EC) (71, 72). For advanced gastric cancer, a single-arm phase II trial (EPOC1706, NCT03609359) showed that 20 of 29 (69%) patients treated by lenvatinib plus pembrolizumab had an objective response and no serious treatment-related adverse events occurred (71). For advanced EC, patients treated by lenvatinib plus pembrolizumab had an ORR at 24 weeks of 38.0%, as shown in a phase Ib/II study (NCT02501096) (72). Importantly, in patients with microsatellite instability (MSI)-high tumors, the ORR at 24 weeks was as high as 63.6% (72). In the phase III trial (NCT03517449), patients with advanced EC were assigned to receive either lenvatinib plus pembrolizumab or physician’s choice chemotherapy in a 1:1 ratio (73). Lenvatinib plus pembrolizumab obtained a longer PFS and OS comparing to chemotherapy in all patients (PFS: 7.2 vs 3.8 months, HR=0.56, 95%CI: 0.47-0.66, p<0.001; OS: 18.3 vs 11.4 months, HR=0.62, 95% CI: 0.51-0.75, p<0.001) (73).

The results from a multicenter phase II basket trial (NCT03827837) showed that the camrelizumab plus famitinib exhibited antitumor activity in patients with platinum-resistant ovarian cancer (OC) (74). At the cut-off date, 24.3% of patients achieved CR or PR. The median PFS and OS of this group of patients were 4.1 months and 18.9 months, respectively (74). Sintilimab plus anlotinib was demonstrated efficacy as second-line or later therapy for patients with PD-L1-positive advanced cervical cancer in a phase II trial, with an ORR of 54.8% (23/42) (75). In several phase II trials, camrelizumab and apatinib combination showed potenial anti-tumor activity and acceptable toxicity for the treatment of advanced cervical cancer (76), triple-negative breast cancer (TNBC) (77), extensive-stage small-cell lung cancer (SCLC) (78), and advanced osteosarcoma (79). The results of these studies are warranted to be validated by larger randomized controlled trials (RCTs).

The activity of bevacizumab plus PD-(L)1 inhibitors has been evaluated in the gynecological tumors. A phase II trial (NCT02873962) showed that nivolumab plus bevacizumab had potential efficacy in patients with relapsed ovarian cancer OC, especially in the platinum-sensitive setting (80). However, the data from a phase III trial (NCT03038100) did not support the use of atezolizumab in newly diagnosed OC (81). In the OS(ITT) population, the median PFS was 19.5 months in patients treated with atezolizumab plus bevacizumab and platinum-based chemotherapy versus 18.4 months in patients treated with placebo plus bevacizumab and platinum-based chemotherapy, respectively (HR=0.92, 95%CI: 0.79-1.07, p=0.28) (81). Furthermore, a phase II trial (NCT02921269) showed that bevacizumab plus atezolizumab did not improve the ORR in cervical cancer (82).

### Conclusions and future directions

In conclusion, the therapy that dual inhibition of VEGF/VEGFR and PD-(L)1, through modulation of both the tumor vasculature and the TIME, has become a key strategy to treat cancer with promising prospect. **Table 1** shows selected phase III trials involving VEGF/VEGFR inhibitors combined with anti-PD-(L)1 agents in solid tumors. In several tumor types, this combination therapy increased patient’s survival and response rate beyond the standard treatment, especially in RCC and HCC. However, not all the tumor types are response to this combination, which may be associated with different TIME in different tumor types (1). The addition of other therapies into this combination may help VEGF/VEGFR inhibitors to enhance PD-(L)1 therapeutic effect. And the tolerable safety profile of this combination supports this hypothesis. Furthermore, VEGF/VEGFR inhibitors plus anti-PD-(L)1 agents can not only be studied in advanced/metastatic disease, but also in the neoadjuvant or adjuvant settings. For example, axitinib plus avelumab as neoadjuvant therapy in patients with non-metastatic RCC has been investigated in a phase II trial (NCT03341845). The preliminary results of this ongoing trial has been reported in the 2022 ASCO Genitourinary Cancers Symposium. For non-metastatic high-risk RCC, neoadjuvant avelumab plus axitinib has been demonstrated to lead to PR of the primary tumor in 30% which is associated with DFS (83).

**Table 1:** Selected phase III clinical trials involving VEGF/VEGFR inhibitors combined with anti-PD-(L)1 agents.

VEGF/VEGFR inhibitor	Anti-PD-(L)1 agent	Treatment	Patient population	Clinical trial ID	Results
Bevacizumab	Atezolizumab	Bevacizumab + atezolizumab (n=454) vs. sunitinib (n=461)	Metastatic RCC	NCT02420821	PD-L1-positive population: HR for PFS = 0.74 (11.2 vs. 7.7 months), p = 0.0217 ITT population: HR for OS = 0.93
Axitinib	Avelumab	Avelumab + axitinib (n=442) vs. sunitinib (n=444)	Clear cell RCC	NCT02684006	PD-L1-positive population: HR for PFS = 0.61 (13.8 vs. 7.2 months), p < 0.001 ITT population: HR for PFS = 0.69 (13.8 vs. 8.4 months), p < 0.001
Axitinib	Pembrolizumab	Pembrolizumab + axitinib (n=432) vs. sunitinib (429)	Clear cell RCC	NCT02853331	ITT population: HR for OS = 0.68 (not reached vs. 35.7 months), p = 0.0003; HR for PFS = 0.71 (15.4 vs. 11.1 months), p < 0.0001
Lenvatinib	Pembrolizumab	Pembrolizumab + lenvatinib (n=355) vs. lenvatinib **+** everolimus (n=357) vs. sunitinib (n=357)	Advanced RCC	NCT02811861	Pembrolizumab + lenvatinib vs. sunitinib: HR for PFS = 0.39 (23.9 vs. 9.2 months), p < 0.001; HR for OS = 0.66, p = 0.005
Cabozantinib	Nivolumab	Nivolumab + cabozantinib (n=323) vs. sunitinib (n=328)	Advanced RCC	NCT03141177	HR for PFS = 0.51 (16.6 vs. 8.3 months), p < 0.001; HR for 12 months survival = 0.60 (85.7% vs. 75.6%), p = 0.001
Bevacizumab	Atezolizumab	Atezolizumab + bevacizumab (n=336) vs. sorafenib (n=165)	Unresectable HCC	NCT03434379	ITT population: HR for PFS = 0.59 (6.8 vs. 4.3 months), p < 0.001; HR for death = 0.58 (OS at 12 months: 67.2% vs. 54.6%), p < 0.001
Bevacizumab biosimilar (IBI305)	Sintilimab	IBI305 + sintilimab (n=380) vs. sorafenib (n=191)	Unresectable HCC	NCT03794440	HR for PFS = 0.56 (4.6 vs. 2.8 months), p < 0.0001; HR for OS = 0.57 (not reached vs. 10.4 months), p < 0.0001
Lenvatinib	Pembrolizumab	Pembrolizumab + lenvatinib (n=411) vs. chemotherapy (n=416)	Advanced endometrial cancer	NCT03517449	pMMR population: HR for PFS = 0.63 (6.6 vs. 3.8 months), p < 0.001; HR for OS = 0.68 (17.4 vs. 12.0 months), p < 0.001; ITT population: HR for PFS = 0.56 (7.2 vs. 3.8 months), p < 0.001; HR for OS = 0.62 (18.3 vs. 11.4 months), p < 0.001;
Lenvatinib	Pembrolizumab	Pembrolizumab + Lenvatinib vs. Pembrolizumab + placebo	Recurrent or Metastatic HNSCC	NCT04199104	Ongoing
Lenvatinib	Pembrolizumab	Pembrolizumab + Lenvatinib vs. SOC	Metastatic Colorectal Cancer	NCT04776148	Ongoing
Apatinib	Camrelizumab	Camrelizumab + apatinib vs. Active surveillance	HCC (High risk of recurrence after curative resection or ablation)	NCT04639180	Ongoing
Apatinib	Camrelizumab	Camrelizumab + apatinib vs. Paclitaxel or Irinotecan	GC/GEJC	NCT04342910	Ongoing
Famitinib	Camrelizumab	Camrelizumab + famitinib vs. pembrolizumab vs. camrelizumab	NSCLC	NCT05042375	Ongoing
Bevacizumab	Atezolizumab	Bevacizumab + atezolizumab vs. TACE	HCC	NCT04803994	Ongoing

RCC, renal cell carcinoma; HR, hazard ratio; PFS, progression-free survival; ITT, intent-to-treat; OS, overall survival; HCC, hepatocellular carcinoma; HNSCC, head neck squamous cell carcinoma; GC, gastric cancer; GEJC, gastroesophageal junction adenocarcinoma; NSCLC, non-small-cell lung cancer.

The bispecific antibodies targeting both VEGF/VEGFR and PD-(L)1 pathways is a promising strategy for cancer treatment and is under development. Cui et al. have developed a novel anti-PD-L1/VEGFR bispecific antibody (HB0025) (84). Preclinical data showed that HB0025 could inhibit two pathways concurrently to enhance its anti-cancer activities, supporting further clinical studies (84). Xiong et al. also generated a non-immunogenic, bispecific antibody targeting both VEGF165 and PD-1, serving as a potential anti-tumor agent (85). And the safety and efficacy of another anti-PD-1/VEGF bispecific antibody AK112 are assessed in the several phase I/II trials (NCT04047290, NCT05116007, and NCT04900363). The initial results from study NCT04047290 demonstrated that AK112 had an encouraging anti-tumor activity and a favorable safety profile in patients with platinum-resistant refractory OC (86).

Oral administration offers more convenience to patients. Currently, there are many oral TKIs targeting VEGF/VEGFR available in market, such as lenvatinib, apatinib and famitinib. However, oral immune PD-(L)1 programs are still in their infancy. In comparison with monoclonal antibodies, small-molecule PD-(L)1 inhibitors could overcome several shortcomings of monoclonal antibodies, such as low tumor penetration and high manufacturing costs (87). More importantly, small-molecule PD-(L)1 inhibitors possess the ability to manage immune-related AE due to their shorter pharmacokinetic exposure (88, 89). To our great delight, several oral small-molecule PD-(L)1 inhibitors are under investigation in clinical trials, such as INCB086550 (NCT04629339, NCT04674748 and NCT05101369), CA-170 (NCT02812875), and IMMH-010 (NCT04343859). The combined administration of both VEGF/VEGFR and PD-(L)1 inhibitors orally is worth waiting for.

## Clinical trials about the dual inhibition of TGF-β and PD-(L)1

### Treatment strategies for the combination of anti-TGF-β and anti-PD-(L)1 agents

The agents inhibit TGF-β pathway usually through the following 3 mechanisms: first, inhibition of TGF-β synthesis directly using antisense molecules such as AP12009; second, blocking TGF-β and its receptors by antibodies or soluble TGF-β decoy receptors (traps) such as fresolimumab (GC1008) and lerdelimumab; third, inhibition of the TGF-β by TKI such as galunisertib (LY2157299) (90).

Currently, several TGF-β inhibitors are under investigation in combination with anti-PD-(L)1 agents. However, the published data are limited. Galunisertib is an oral TKI of type I TGFβ receptor (TGFβRI). Galunisertib plus anti-PD-L1 antibody durvalumab has been assessed in metastatic pancreatic cancer in a phase I trial (NCT02734160) (91). The results showed that galunisertib plus durvalumab had an acceptable tolerability, but clinical activity was limited with an ORR of 3.1% and a DCR of 25.0% (91). The poor efficacy may due to the aggressive nature of pancreatic cancer, as ICIs in pancreatic cancer has shown minimal response in previous studies (91). The safety profile of galunisertib plus durvalumab could lend to this treatment protocol in combination with other treatments in future trials. The combination of galunisertib and anti-PD-1 antibody nivolumab has also been evaluated in a phase I/II trial in patients with solid tumors including recurrent NSCLC and HCC (NCT02423343). The trial has been completed, however, the results have not been published. LY3200882 is a novel, selective TGFβRI inhibitor. Using LY3200882 to inhibit TGFβ and LY3300054 to inhibit PD-L1 has been assessed in solid tumors in a phase I trial (NCT02937272) (92). The efficacy of this combination did not meet the expectations, with an ORR of 7.7% (1/13) and DCR of 39% (5/13) (92). Gemogenovatucel-T is an autologous tumor cell vaccine which specifically decreases the expression of TGF-β1 and TGF-β2 (93). The anti-tumor activity of Gemogenovatucel-T plus aezolizumab (NCT03073525) or durvalumab (NCT02725489) are under investigation in patients with advanced gynecological cancers. SAR439459 is a pan-TGFβ neutralizing antibody (94). In a phase I trial (NCT03192345), the anti-tumor activity of SAR439459, either alone or in combination with PD-1 inhibitor cemiplimab, are under investigation. Vactosertib is a selective small-molecule inhibitor of TGF-βRI (95). Vactosertib plus PD-1 inhibitor pembrolizumab is under investigation in NSCLC (NCT04515979) and metastatic colorectal or gastric cancer (NCT03724851). NIS793 is an anti-TGFβ antibody. The phase I trial (NCT02947165) about the safety of NIS793 as a single agent and in combination with spartalizumab (PDR001, anti-PD-1 antibody) in advanced tumors has been completed. However, the data are not published. Furthermore, the efficacy of NIS793 plus spartalizumab (PDR001) plus SOC in metastatic pancreatic ductal adenocarcinoma (mPDAC) in first-line setting is under investigation in a phase II trial (NCT04390763).

Taken together, most of the clinical trials about anti-TGF-β plus anti-PD-(L)1 agents in solid tumors are still under investigation. From the published data, we could found that this combination provided a manageable safety profile. However, the clinical activity observed with this combination strategy is limited. Some studies have suggested that development of a dual-targeting agent that localize TGF-β inhibition within the TME may be a possible improvement for this situation (12).

### Bispecific antibodies against TGF-β and PD-(L)1

**Figure 1:**
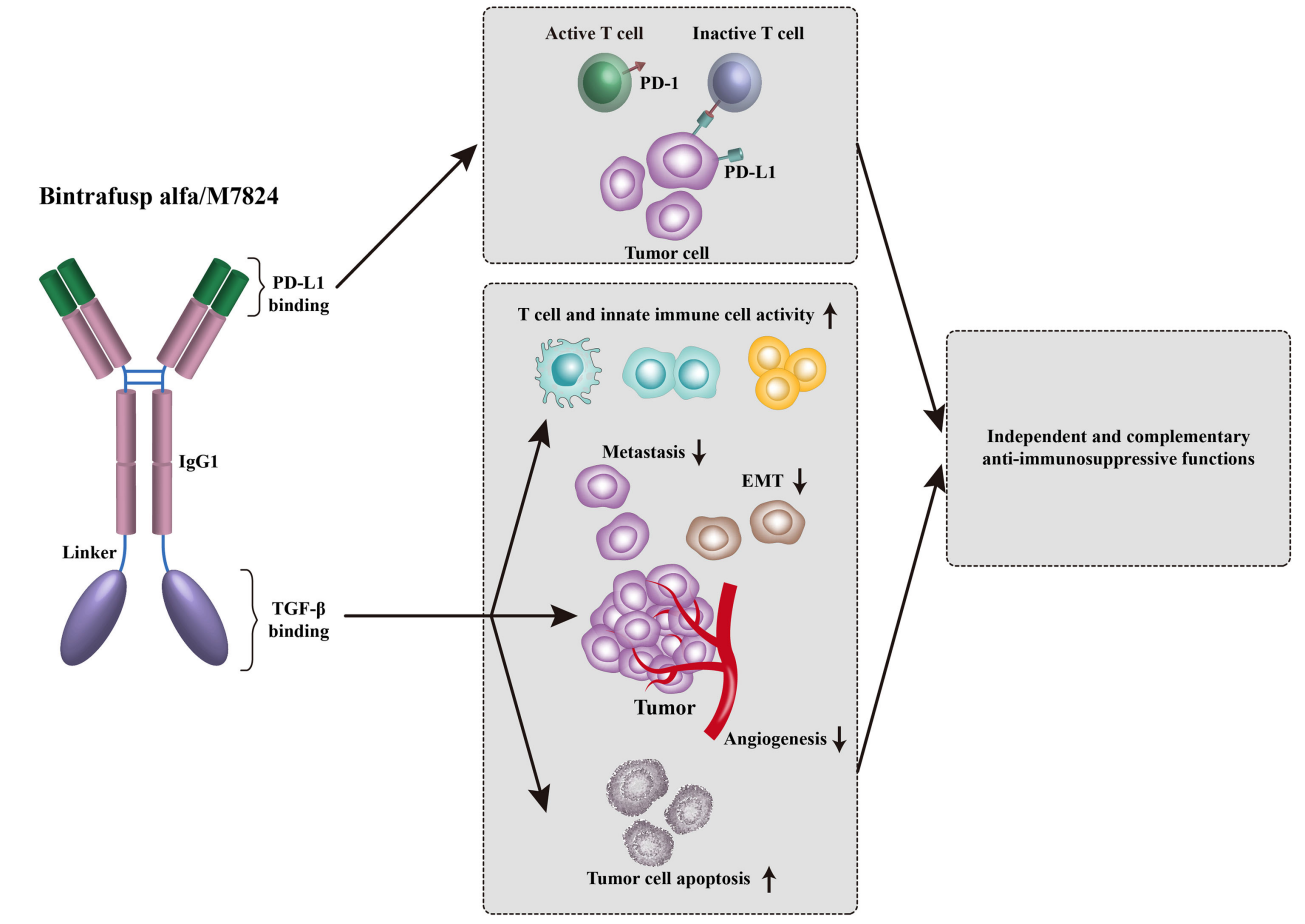
Bintrafusp alfa was designed to simultaneously inhibit PD-1/PD-L1-mediated immunosuppression while decreasing the levels of TGF-β in the TME *via* a TGF-β ‘trap’ portion.

Development of bispecific antibody targeting both TGF-β and PD-(L)1 is a hotspot of tumor immunotherapy. Bintrafusp alfa, previously known as M7824, is a bifunctional fusion protein against TGF-βRII and PD-L1 (**Figure 1**). Preclinical studies showed that bintrafusp alfa reduced TGF-β signaling in TME, resulting in greater tumor volume decrease compared with anti-PD-L1 antibody alone (96). Nineteen patients with advanced solid tumors were treated with bintrafusp alfa in a phase I trial (NCT02517398) (97). In this study, bintrafusp alfa exhibited a manageable safety profile, which was similar to anti-PD-1 monotherapies (97). For efficacy, 1 of 19 patients enrolled experienced a durable CR and 2 patients had a durable PR (97). In the expansion cohort of pretreated advanced NSCLC (NCT02517398), bintrafusp alfa treatment showed an encouraging efficacy with an ORR of 21.3% (17/80) in all patients and 85.7% (6/7) in patients with PD-L1-high (11). In the expansion cohort of heavily pretreated SCCNH (NCT02517398), 4 out of 32 patients received bintrafusp alfa achieved PR (98). Fifty-nine patients with advanced, pretreated human papillomavirus (HPV)-associated cancers (including anal cancer, cervical cancer, and oropharyngeal cancer) from trials of NCT02517398 and NCT03427411, received bintrafusp alfa treatment (99). For full-analysis population, the ORR was 30.5% (including 5 CR) and DCR was 44.1%, supporting further investigation of bintrafusp alfa in such tumor types (99). Several data of a phase I trial conducted in Asian patients (NCT02699515) have also been published. Twenty-three Asian patients (including 9 HCC patients) had received bintrafusp alfa, among them, 8 experienced treatment-related adverse events (TRAEs) (100). In an expansion cohort of advanced gastric/gastroesophageal junction cancer, 31 heavily pretreated patients who received bintrafusp alfa achieved an ORR of 16% and a DCR of 26% (101). Bintrafusp alfa also showed clinical activity in Asian patients with pretreated biliary tract cancer (BTC) in the trial of NCT02699515 (102). The ORR was 20%, with 5 of 6 responses ongoing at the data cut-off. These data prompted progression into phase II and III trials in BTC (NCT03833661 and NCT04066491). Furthermore, the safety profile of bintrafusp alfa shown in two phase I trials (NCT02699515 and NCT02517398) is manageable, with the most common TRAEs of rash maculopapular, pruritus, rash, asthenia, and hypothyroidism. The potential therapeutic effects and safety profile of bintrafusp alfa shown in phase I trials have led to further investigation of bintrafusp alfa.

However, the following phase II and phase III trials failed to meet their primary end points, casting a deep gloom over the development of bintrafusp alfa. In March 2021, the phase II INTR@PID BTC 047 trial (NCT03833661), which examined the efficacy of second-line treatment with bintrafusp alfa for 159 BTC patients, was announced to be failed (103). In August 2021, the phase II/III INTR@PID BTC 055 trial (NCT04066491) was announced to be discontinued based on a review of the data conducted by the Independent Data Monitoring Committee (IDMC), which concluded the trial was unlikely to meet its primary objective (103). In addition, Bintrafusp alfa monotherapy has also shown disappoint results comparing to pembrolizumab in the first-line treatment of patients with NSCLC (NCT03631706) (103). Based on the recommendation of the IDMC, the sponsor decided to discontinue the trial. The disappointed results mentioned above did not lead to the sponsor to give up the development of bintrafusp alfa completely. Currently, over 30 clinical trials about bintrafusp alfa are ongoing or actively recruiting patients according to ClinicalTrials.gov.

Although the disappointed data about bintrafusp alfa in BTC and NSCLC have been revealed, the enthusiasm of other companies to develop TGFβ–PD-(L)1 bifunctional fusion protein have not been reduced. Other PD-(L)1/TGF-β bispecific antibodies, such as SHR-1701, AVID200, TQB2858, TQB2868, PM8001, YM1001 and JS201, are in development. SHR-1701 is a novel bifunctional anti-PD-L1/TGF-βRII agent. The preliminary results of a phase I trial of SHR-1701 (NCT03710265) reported in 2021 ASCO showed that SHR-1701 monotherapy had an acceptable safety profile, no DLT was observed (104). The ORR was 17.8%, with 8 patients achieving PR (104). In the same period, the results from an expansion cohort of a phase I study (NCT03774979) were also reported. SHR-1701 showed encouraging antitumor activity in patients with advanced EGFR-positive NSCLC after failure of at least one line standard EGFR TKI treatment, with an ORR of 16.7% and a DCR of 50.0% (105). SHR-1701 also showed encouraging antitumor activity and controllable safety in patients with pretreated advanced cervical cancer (NCT03774979) (106), pretreated recurrent/refractory (r/r) gastric cancer (NCT03710265) (107), and treatment-naive PD-L1 positive advanced/metastatic NSCLC (NCT03774979) (108). Currently, several clinical trials about SHR-1701 are ongoing. Due to the manageable safety profile of SHR-1701, the combination of SHR-1701 and other therapies has been applied in clinical trials to obtain better clinical efficacy. For example, NCT05020925 is a phase I/II trial to assess the efficacy and safety of SHR-1701 in combination of famitinib in patients with recurrent/metastatic nasopharyngeal carcinoma; NCT05179239 is a phase III trial that evaluates the anti-tumor activity of SHR-1701 or placebo plus chemotherapy with or without BP102 (a bevacizumab biosimilar) patients with recurrent/metastatic cervical cancer. **Table 2** summarized the ongoing phase II/III clinical trials of SHR-1701 according to ClinicalTrials.gov.

**Table 2:** Ongoing phase II/III trials of SHR-1701.

Treatment	Disease	Phase	Estimated enrollment	Clinical trial ID
SHR-1701 + chemotherapy + BP102	Cervical cancer	III	572	NCT05179239
SHR-1701 + chemotherapy + bevacizumab	Non-squamous NSCLC	III	561	NCT05132413
SHR-1701 + chemotherapy	GC/GEJC	III	920	NCT04950322
SHR-1701 + chemotherapy + BP102	mCRC	II/III	439	NCT04856787
SHR-1701 + temozolomide	Melanoma	II	31	NCT05106023
SHR-1701	HNSCC	II	130	NCT04650633
SHR-1701 + fluzoparib	Advanced/metastatic NSCLC	II	71	NCT04937972
SHR-1701 + BP102	Non-squamous NSCLC	II	71	NCT04974957
SHR-1701+ Famitinib	SCLC	II	106	NCT04884009
SHR-1701+ Famitinib	Advanced/metastatic NSCLC	II	168	NCT04699968
SHR-1701+ Famitinib	Recurrent/metastatic NPC	II	30	NCT05020925
SHR-1701 + chemotherapy	Pancreatic cancer	I/II	56	NCT04624217

NSCLC, non-small-cell lung cancer; GC, gastric cancer; GEJC, gastroesophageal junction adenocarcinoma; mCRC, metastatic colorectal cancer; HNSCC, head neck squamous cell carcinoma; SCLC, small-cell lung cancer; NPC, nasopharyngeal carcinoma; BP102, a bevacizumab biosimilar; Fluzoparib, a PARP inhibitor; Famitinib, a multitargeted TKI tyrosine kinase inhibitor.

### Conclusions and future directions

TGF-β is an important immune regulator that allows cancer cells to escape immune surveillance (109). Clinical trials have tried to combine anti-TGF-β and anti-PD-(L)1 agents in treating solid tumors. However, the clinical activity observed with this combination strategy is not satisfactory from the published data. A bispecific antibody targeting both pathways, such as bintrafusp alfa and SHR-1701, has the potential for localizing inhibition of TGF-β within the TME, a benefit that is not provided by combining the two independent therapies. The bispecific antibodies show potential therapeutic effects for various tumor types with a manageable safety profile. However, no head-to-head clinical trial has confirmed that the bispecific antibodies against both TGF-β and PD-(L)1 pathways have significant advantages over anti-PD-(L)1 monotherapies or combinations of TGF-β and PD-L1 independent therapies. Furthermore, the biology of TGFβ in TME is not very clear now. Whether targeting TGFβ will enhance the efficacy of cancer immunotherapy should be further confirmed. And better knowing the biology of TGFβ will facilitate the selection of patients that most likely to be benefited from this combination therapy. The manageable safety profile of bispecific antibody against TGF-β and PD-(L)1 allows this therapy plus other modalities such as targeted therapy, chemotherapy and radiation therapy as the SOC potentially in traditionally “cold” tumors. These combinations can not only be studied in advanced/metastatic disease, but also in the adjuvant setting after better knowing the biology of TGFβ.

## Clinical trials about the triple inhibition of VEGF/VEGFR, TGF-β and PD-(L)1

It has been reported that TGF-β and VEGF cooperatively control the immunosuppressive TME (13). And the combination of VEGF/VEGFR inhibitors and anti-PD-(L)1 agents or the dual inhibition of TGF-β and PD-(L)1 has shown potential efficacy and manageable safety profile in clinical trials, thus, the triple inhibition of VEGF/VEGFR, TGF-β and PD-(L)1 seems rational. Currently, some clinical trials have attempted to combine PD-(L)1/TGF-β bispecific antibodies and VEGF/VEGFR inhibitors in solid tumors, especially in difficult-to-treat tumors including BTC, pancreatic cancer and SCLC. These studies are all ongoing or in actively recruiting patients status, with a few preliminary results being reported.

The preliminary results of an ongoing exploratory phase II study (ChiCTR2000037927) evaluating the safety and efficacy of SHR-1701 in combination with famitinib in advanced pancreatic cancer and BTC patients who have failed previous standard treatment have been revealed in the 2022 ASCO Gastrointestinal Cancers Symposium (110). Up to 15 Sep 2021, 15 and 9 patients were enrolled in pancreatic cancer and BTC cohorts, respectively. In the pancreatic cancer cohort, the ORR (1 CR and 1 PR) and DCR were 13% and 53%, respectively (110). Among 8 evaluable patients in BTC cohort, the ORR (1 PR) and DCR were 13% and 63%, respectively (110). The most frequently reported TRAEs were proteinuria (58%), hypertension (42%), and blood urine present (42%) (110). These results indicated that HR-1701 plus famitinib had encouraging activity with manageable safety in advanced pancreatic cancer or BTC. SHR-1701 in combination of famitinib in other solid tumors, such as recurrent/metastatic nasopharyngeal carcinoma (NCT05020925), extensive stage SCLC (NCT04884009), advanced or metastatic NSCLC (NCT04699968), are also under investigation. Similarly, SHR-1701 is also attempted to be combined with a bevacizumab biosimilar BP102 in the treatments of solid tumors (NCT04974957, NCT04856787 and NCT05179239).

The efficacy and safety of JS-201, a PD-L1/TGF-β bispecific antibody, combined with lenvatinib in the treatment of SCLC that has failed previous chemotherapy combined with anti-PD-L1 are under investigation in a phase II trial (NCT04951947). TQB2858 is another PD-L1/TGF-β bispecific antibody developed in China. TQB2858 in combination with anlotinib is planned to be evaluated in advanced EC (NCT05121363), recurrent/metastatic nasopharyngeal cancer (NCT05198531) and advanced pancreatic cancer (NCT05193604).

The inhibition of VEGF/VEGFR and TGF-β pathways can overcome microenvironmental resistance to PD-1 blockade (13). Thus, in theory, triple inhibition of VEGF/VEGFR, TGF-β and PD-(L)1 would be a promising strategy to overcome treatment resistance of immunotherapy. Currently, this treatment strategy is under investigation in clinical trials with a bit of data being reported. We await, with interest, more data from the ongoing clinical trials will support the application of this approach in patients with cancers. We also believe that more convenient and effective strategies will be developed in the future to inhibit VEGF/VEGFR, TGF-β and PD-(L)1 pathways synergistically. However, under the condition that the efficacy of the dual inhibition of TGF-β and PD-(L)1 has not been determined, whether triple inhibition of VEGF/VEGFR, TGF-β and PD-(L)1 will have promising efficacy especially in difficult-to-treat tumors is still a problem needed to be solved.

## Conclusions

In this review, we described the existing preclinical and clinical evidence supporting the use of anti-VEGF and/or anti-TGF-β therapies to enhance cancer immunotherapy. VEGF/VEGFR and TGF-β pathways play an important role in the development and function of the TME, contributing to the immunosuppressive status of TME. To inhibit VEGF/VEGFR and/or TGF-β pathways can restore TME from immune-suppressive to immune-supportive status and enhance sensitivity to anti-PD-(L)1 treatment. Encouragingly, adopting anti-VEGF and/or anti-TGF-β therapies as a combination treatment with anti-PD-(L)1 therapy have been demonstrated as effective and tolerable in several solid tumors in clinical trials. There is no doubt that anti-VEGF and/or anti-TGF-β therapies have opened a new window for cancer immunotherapy. The clinical value of this combination strategy is worthy to be studied further.

## Author contributions

RD designs this review. LL and RD wrote the text and collected related data. QW is responsible for revising the content of the article. All authors contributed to the article and approved the submitted version.

## Funding

This work was supported by the Science and Technology Project of the Health Planning Committee of Sichuan Province (No. 21PJ092).

## Conflict of interest

The authors declare that the research was conducted in the absence of any commercial or financial relationships that could be construed as a potential conflict of interest.

## Publisher’s note

All claims expressed in this article are solely those of the authors and do not necessarily represent those of their affiliated organizations, or those of the publisher, the editors and the reviewers. Any product that may be evaluated in this article, or claim that may be made by its manufacturer, is not guaranteed or endorsed by the publisher.
